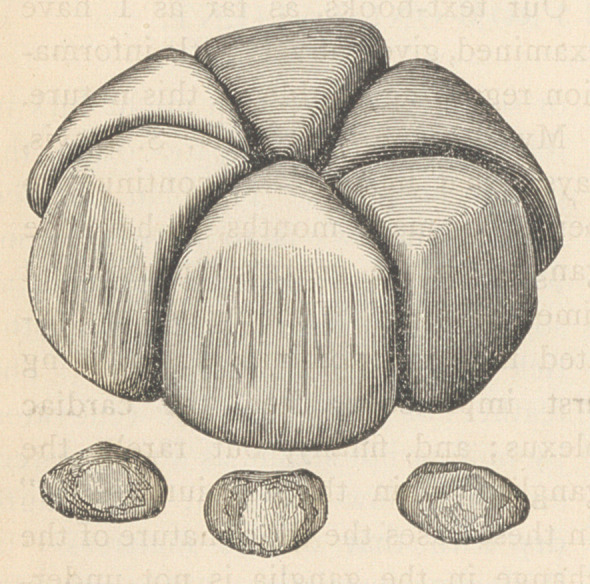# Nine Calculi Taken from One Patient

**Published:** 1874-06-15

**Authors:** 


### NINE CALCULI TAKEN FROM ONE PATIENT.

DURING the recent visit of the members of the State Medical Society to Mercy Hospital, Prof. E. Andrews brought before them a newly entered patient, suspected of having vesical calculus. He introduced a sound, and proved by the audible click that the suspicion was well founded. The sensation communicated by the instrument also proved that the case was multiple, and the stones of pretty large size. The patient was about fifty years of age, but in fair health, and the bladder not much inflamed. The large quantity of calculous material present, and the great apparent hardness of the stones, judged by the sharpness of the click elicited, when struck, determined the case to be suitable for lithotomy rather than lithotrity. The patient was then placed on a free use of tinct. of iron for a few days, to prepare his system for the operation, and assigned a well-ventilated ward, where his portion of the air-space amounted to 3,000 cubic feet. He was placed on a new mattress and given a pillow, whose feathers had been renovated by hot steam, and the tick washed, that no pyæmic or septicemic infection might lurk in the bedding, as it is too often the case in hospitals. The plan pursued by Dr. Andrews is to have the contents of every mattress burned as soon as the patient leaves it, while the tick is sent to the laundry. The feathers of the pillows are renovated by boiling hot steam, as above stated, and the pillow-ticks sent to the laundry with that of the mattress. In this way every patient comes virtually upon a new mattress, and upon pillows as pure as new ones.

While preliminarily taking the iron for a few days, the patient was ordered good nourishing diet, and directed to spend a considerable portion of his time out of doors, but not to undertake any violent exercises.

On May 25, he was placed on the table and anesthetized with sulphuric ether. Prof. Andrews then proceeded to perform lithotomy by the usual lateral method. The first introduction of the forceps brought away a smooth stone over an inch in diameter, with three large facets upon it, showing that there were other calculi present. The second trial drew out two smaller stones. The third brought away another large one; the fourth, fifth, sixth, etc., had the same result until nine calculi had been extracted, six of which were over one inch each in diameter. The combined weight of the nine stones was five and a half avoirdupois ounces. They were all smoothed by attrition, and rendered somewhat triangular by the arrangement of the flattened faces, that rested against each other, so that the six larger ones fitted together in a sort of circle, as shown in the accompanying engraving, which doubtless indicates the way they lay in the bladder. The three smaller stones were likewise more or less angular in form, and polished. The engraving is about two-thirds of the actual diameter.

Twenty-four hours after the operation, the patient (who had lived in a malarious district) had a severe chill, followed by some hours of fever, and closing with a copious sweat. Vigorous doses of quinine prevented any repetition of the chills, and tincture of iron was given in addition five times a day.

On the tenth day some enteric disturbance, resembling that of typhoid fever, occurred, and the treatment was changed to nitric acid and strychnia. At present (thirteenth day), this complication still exists, with a slight fever and some want of moisture on the tongue. The wound looks well, but the temperature of the body, tested in the axilla, is over 101 degrees. There are no pyæmic chills nor sweats.

June 10th—Patient doing well.

## Figures and Tables

**Figure f1:**